# Dairy consumption and its association with anthropometric measurements, blood glucose status, insulin levels, and testosterone levels in women with polycystic ovary syndrome: a comprehensive systematic review and meta-analysis

**DOI:** 10.3389/fendo.2024.1334496

**Published:** 2024-10-03

**Authors:** Hadith Rastad, Ehsan Shahrestanaki, Hamid Reza Heydarian, Mina Maarefvand

**Affiliations:** ^1^ Cardiovascular Research Center, Alborz University of Medical Sciences, Karaj, Iran; ^2^ Department of Epidemiology, School of Public Health, Iran University of Medical Science, Tehran, Iran; ^3^ Non-Communicable Diseases Research Center, Alborz University of Medical Sciences, Karaj, Iran; ^4^ Social Determinants of Health Research Center, Alborz University of Medical Sciences, Karaj, Iran; ^5^ Evidence-based Phytotherapy and Complementary Medicine Research Center, Alborz University of Medical Sciences, Karaj, Iran

**Keywords:** dairy consumption, polycystic ovary syndrome (PCOS), anthropometric measurements, blood glucose, insulin levels, women, systematic review, meta-analysis

## Abstract

**Background:**

We conducted a systematic review and meta-analysis on dairy consumption and its association with anthropometric measurements, blood glucose status, insulin levels, and testosterone levels in women with Polycystic Ovary Syndrome.

**Methods:**

This study conducted a comprehensive literature search using electronic databases like MEDLINE, Scopus, PubMed, Web of Science, and Google Scholar to identify observational and interventional studies investigating the relationship between dairy product consumption and Polycystic Ovary Syndrome. A meta-analysis was performed on clinical trial studies that examined the effect of a low starch/low dairy diet in Polycystic Ovary Syndrome subjects. Statistical analyses were performed using Stata version 16.0 (Stata Corporation, College Station, Texas, USA), and statistical significance was defined as p-value < 0.05.

**Results:**

Of the 1,313 citations reviewed, our systematic review identified 11 studies that met the inclusion criteria, comprising six case-control studies, four clinical trials, and one cross-sectional study. The case-control studies found limited evidence of an association between dairy consumption and Polycystic Ovary Syndrome. The result of the clinical trial studies in meta-analysis showed that reducing dairy intake along with reducing starch intake led to statistically significant improvements in anthropometric and metabolic measures including mean weight (Standardized mean difference: -8.43 (95% CI: -9.01, -7.86)), Body mass index (-3.14 (95% CI: -3.35, -2.92), waist circumference (-6.63 (95% CI: -10.70, -2.57)) and Waist-to-Height Ratio (-0.04 (95% CI: -0.07, -0.01), insulin fasting (-18.23 (95% CI: -22.11, -14.36)), insulin 120 minutes (-94.05 (95% CI: -157.67, -30.42)), HbA1c (-0.27 (95% CI: -0.37, -0.17)), Ferryman-Gallwey score (-2.07 (95% CI: -2.98, -1.16)) and total testosterone (-9.97 (95% CI: -14.75, -5.19)). No significant reduction was found in fasting glucose, 2 hours glucose, percent of fat mass, and mean free testosterone after intervention.

**Conclusions:**

The findings of this systematic review show limited evidence about the association between dairy consumption and Polycystic Ovary Syndrome. The interventional studies suggest that a low-dairy/low-starch diet may improve some anthropometric and metabolic measures in women with Polycystic Ovary Syndrome.

## Introduction

Polycystic ovary syndrome (PCOS) is a common hormonal disorder in women of reproductive age, characterized by irregular menstrual cycles, elevated androgen levels, and polycystic ovaries (1). The prevalence of PCOS varies between ranging from 4% to 18% (2). The wide range of prevalence can be attributed to the complex pathophysiology of PCOS, the variable clinical presentation, and the lack of adequate evidence-based data due to the existence of several diagnostic guidelines (3).

The exact causes of PCOS remain largely unknown, but it is believed that hormonal imbalances, including an excess of androgens and/or insulin, play a role (4). Additionally, environmental factors such as geography, diet, socioeconomic status, and environmental pollutants may also contribute to the development and management of PCOS (5).

Many women with PCOS are also overweight or obese, and weight loss can be challenging due to insulin resistance and compensatory hyperinsulinemia (6). The management of PCOS focuses on improving both the reproductive and metabolic symptoms (7), and lifestyle modifications, such as physical activity and diet, are critical for improving adverse outcomes associated with the condition (8, 9). Specifically, dietary modifications that reduce hyperinsulinemia may help improve fatty acid oxidation, facilitate weight loss, and prevent further weight gain (10).

Recent research has highlighted the potential significance of dairy products in the diet of women with PCOS, particularly in relation to carbohydrate metabolism disorders such as insulin resistance or type 2 diabetes. Dairy products contain valuable nutrients such as calcium, vitamin D, and high-quality proteins, which can have beneficial effects on overall health (11). Studies indicate that the consumption of carbohydrates from dairy and starch-based foods elicits a higher postprandial insulin response compared to carbohydrates from non-starchy vegetables and fruits (12, 13). To evaluate the differences in dairy consumption in women with and without PCOS and the effect of dairy diets on PCOS complications, a comprehensive systematic review and meta-analysis of all available studies was conducted.

## Materials and methods

This study was reported according to the Preferred Reporting Items for Systematic Reviews and Meta-Analyses (PRISMA) statement. This study was approved by the ethics committee of Alborz University of Medical Sciences with the code IR.ABZUMS.REC.1401.061.

All eligible studies that assessed the relationship between dairy consumption and PCOS, were included in this systematic review.

### Search strategy

The review question was formulated using the PECO framework in observational study and PICO in interventional study. Population (P) was women, Exposure (E) in observational study and intervention study (I) was dairy product, Comparison (C) was control group (if applicable) and Outcome (O) was Polycystic Ovary Syndrome. the Search strategy was performed based on PECO and PICO.

A comprehensive literature search was performed in the electronic databases (including MEDLINE/PubMed, Scopus, Web of Science, and Google Scholar) until 17 June 2023 to identify eligible studies investigating the association of PCOS with dairy product consumption. The search was conducted in each database using a combination of the key terms in two domains (1): PCOS and (2) Dairy product (**Table 1**).

**Table 1:** Search strategy.

PubMed
((Polycystic Ovary Syndrome) OR (“Polycystic Ovary Syndrome” [Mesh])) AND ((“Dairy Products” [Mesh]) OR (dairy)) OR (“Milk” [Mesh])) OR (milk)) OR (“Cheese” [Mesh])) OR (Cheese)) OR (“Butter” [Mesh])) OR (butter)) OR (“Buttermilk” [Mesh])) OR (buttermilk)) OR (“Yogurt” [Mesh])) OR (yogurt)) OR (curd)) OR (“Whey” [Mesh])) OR (whey))
Scopus
( ( TITLE-ABS-KEY ( polycystic AND ovary AND syndrome ) OR TITLE-ABS-KEY ( pcos ) OR ( TITLE-ABS-KEY ( polycystic AND ovary AND syndrome ) OR TITLE-ABS-KEY ( pcos ) AND ( (TITLE-ABS-KEY (Dairy Products) OR TITLE-ABS-KEY ( dairy ) OR TITLE-ABS-KEY ( milk ) OR TITLE-ABS-KEY ( cheese ) OR TITLE-ABS-KEY ( butter ) OR TITLE-ABS-KEY ( buttermilk ) OR TITLE-ABS-KEY ( yogurt ) OR TITLE-ABS-KEY ( curd ) OR TITLE-ABS-KEY ( whey ) ( TITLE-ABS-KEY ( dairy )(
ISI/WOS
(Polycystic Ovary Syndrome (All Fields) or (PCOS) AND (dairy (All Fields) or milk (All Fields) or Cheese (All Fields) or butter (All Fields) or buttermilk (All Fields) or yogurt (All Fields) or curd (All Fields) or whey (All Fields))

### Selection process

All retrieved articles were imported into EndNote X8.2, and duplicates were automatically removed from the list. Two researchers independently screened titles, abstracts, and full texts, respectively. Furthermore, they examined the reference list of eligible studies to identify any potentially eligible citations. Disagreements between researchers were resolved by discussing the full texts.

### Eligibility criteria

Our systematic review included interventional studies that examined the effects of dairy product consumption on PCOS-related complications and met the following criteria (1): the intervention involved a dietary intervention with dairy products, and (2) the study participants were women with PCOS. In addition, observational studies comparing the dairy product intake in women with and without PCOS were also included. Studies that were not written in English, as well as letters to the editor and editorials, were excluded from our analysis.”

### Data extraction

Data extraction from the included studies was conducted by two researchers using Microsoft Excel 2013. The following information was extracted: author details, country of origin, study design, sample size (for both intervention and control groups), age of participants (mean and standard deviation (SD)), anthropometric measurements (including weight, Body mass index (BMI), waist circumference (WC), and Waist-to-Height Ratio (WHtR)), blood glucose tests (including fasting glucose, 2-hour glucose, percentage of fat mass, fasting insulin, insulin at 120 minutes, and HbA1c), variables used for confounding adjustment, PCOS diagnosis, eligibility criteria, assessment tool, time to event (in weeks), and effect size (mean and SD). Any discrepancies between the two researchers were resolved through discussion and review of the full-text articles.

### Quality assessment

Two researchers assessed the quality of the studies using the National Institutes of Health (NIH) quality assessment scales for before-and-after studies without control groups, cross-sectional, and case-control studies (14). Any discrepancies between the researchers were resolved through discussion and review of the full-text articles.

### Statistical analysis

To assess heterogeneity among the studies, we used both the Chi-squared and I^2^ tests. A random-effects model was employed if the heterogeneity was statistically significant (Chi-squared; P-value<0.10); otherwise, a fixed-effects model was used. We conducted a meta-analysis of clinical trial studies that investigated the impact of a low starch/low dairy diet on PCOS patients before and after the intervention. For these studies, we summarized the mean and standard deviation using the standard mean difference (SMD) and 95% confidence interval (CI). Meta-analysis was performed for the following endpoints: anthropometric measurements, blood glucose levels, Ferryman-Gallwey score, and testosterone levels. All statistical analyses were conducted using Stata version 16.0 (Stata Corporation, College Station, Texas, USA

## Results

**Figure 1:**
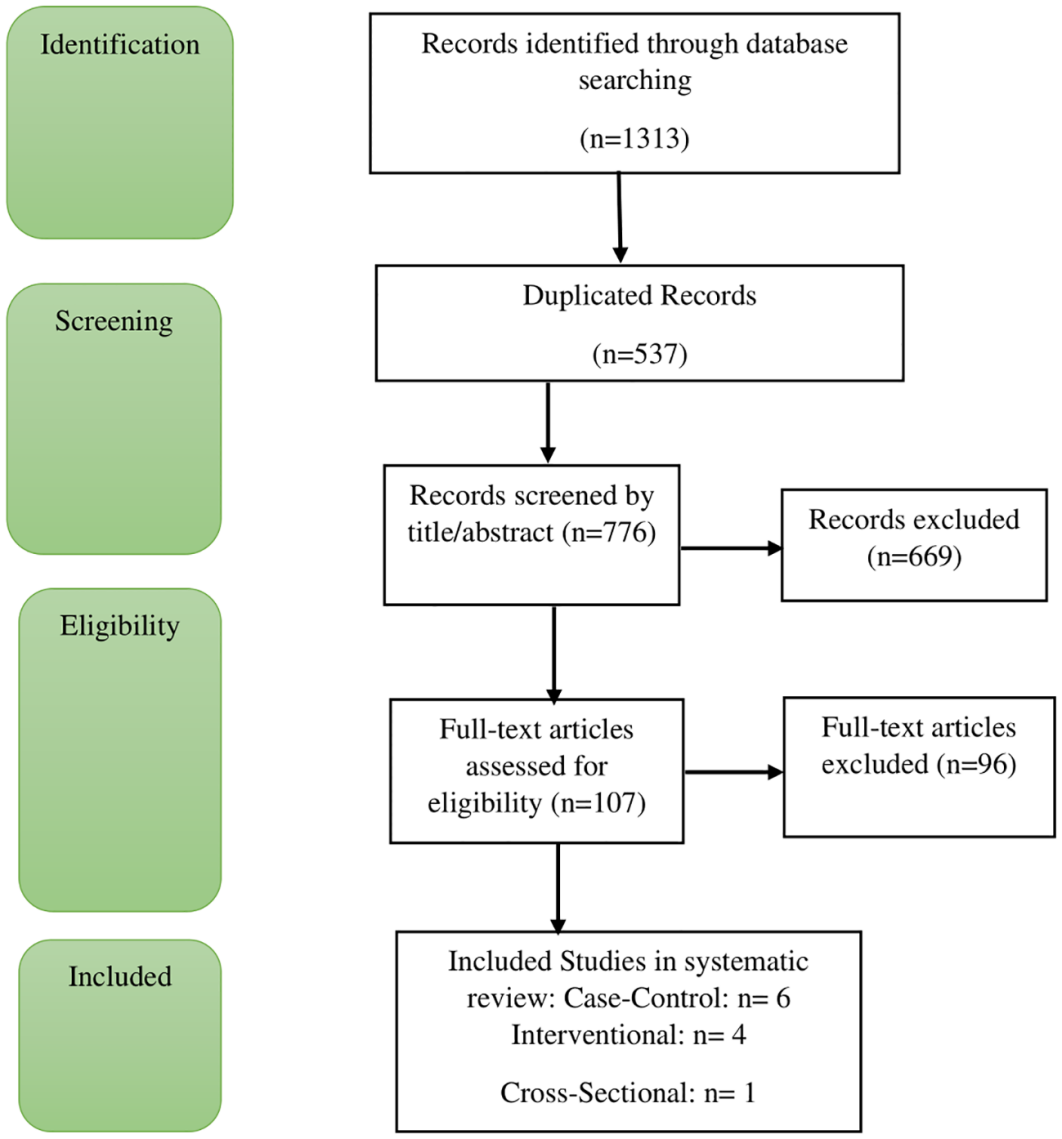
PRISMA flowchart of literature search and selection process. Adapted from PRISMA 2020 flow diagram templates, licensed under CC BY 4.0.

In the initial literature search, a total of 1,313 citations were retrieved from electronic databases, including 50 from PubMed, 320 from Scopus, and 256 from ISI/Web of Science. An additional 687 citations were identified through a manual search on Google Scholar. After removing the duplicates automatically, 776 studies were assessed using titles and abstracts. Then, 107 studies were evaluated for full texts. Finally, 11 articles were found to be relevant to our topic. Out of the 11 studies, 6 were case-control, 4 were clinical trials, and 1 was cross-sectional. The detailed selection process is illustrated in **Figure 1**.

The reasons for excluding the studies were as follows: not investigating the intended outcomes of this review study (n= 68), study design (n=15)), not examining dairy products as a separate item (n= 9), being repetitive (n=3), and using non-English language in the text.

### Case-control studies

#### Description

In this systematic review, six case-control studies were reviewed, of which five studies were conducted in Iran and one study in Italy. A total of 1,011 individuals with PCOS and 887 controls with different diagnostic criteria participated in these studies (**Table 2**).

**Table 2:** **C**haracteristics of the included case-control Studies.

ID	Author	Country	Design	Sample size		Age(year) Mean ± SD		confounders	PCOS diagnosis	Eligibility criteria	Assessment tool
				PCOS	Control	PCOS	Control				
1	Shishehgar et al. (2016) (22)	Iran	CC	142	140	28.5 ± 4.9	28.9 ± 5.8	BMI, age	AES criteria	Non pregnant or not on lactation, without hyper PRL, TD, NCAH, CD, chronic disease (liver, kidney disease, HTN, DM, cancer), Not using of insulin or sensitizing agents, OCP, medication affecting appetite or weight, weight loss diet or exercise	FFQ
2	Hosseini et al. (2017) (19)	Iran	CC	99	198	29.0 ± 5.5	29.2 ± 6.0	BMI, age	AES criteria	-Controls: Healthy women -Case: PCOS without Androgen secreting tumors, congenital adrenal hyperplasia, CD, TD, severe insulin resistance syndrome, DM, Hyper PRL, HTN, CVD, use of androgenic or anabolic drugs	FFQ
3	Badri-Fariman et al. (2021) (20)	Iran	CC	120	120	20-48	20-48	Age, BMI	Rotterdam criteria	-Controls: without PCOS diagnosis, with normal menstrual - Case: PCOS without CVD, liver, and kidney diseases, smoking, taking drugs that can affect the metabolism of hormones and body composition, having strenuous physical activities, not consent to participate	USDAQ
4	Rajaeieh et al. (2014) (23)	Iran	CC	347	38	29.5 ± 4.8	29.5 ± 4.8	–	Clinical D.	Lack of precocious puberty, not having uterus cancer and typical (chronic) diseases, not being pregnant, without specific diet.	FFQ
5	Altieri et al. (2012) (24)	Italy	CC	100	100	27·7 ± 5·2	28·4 ± 5·8	age	Rotterdam criteria	-No other causes of HA such as Hyper PRL, CS, CAH -Exclusion criteria: presence of serious concomitant illness, based on clinical examination and routine laboratory findings, including DM, other endocrine and metabolic disturbances, eating disorders, use of OCP, Insulin-sensitizing, glucose lowering, lipid-lowering or psychoactive medications within 6 months from assessment	7DFQ
6	Zirak Sharkesh et al. (2021) (21)	Iran	CC	203	291	28.98 ± 5.43	30.15 ± 6.21	age	Rotterdam criteria	No history of hypothyroidism, hyper PRL, CS, adrenal hyperplasia, drug use including OCP, hormonal drugs, and glucocorticoids, taking mineral supplements and vitamins, having a special diet for the past 6 months, smoking, alcohol intake, subjects more than a year has passed since their diagnosis of PCOS, pregnant or lactating women underreporting or over reporting of energy Exclusion criteria: intake less than 800 kcal and more than 4200 kcal and not answering more than 40 items in the FFQ	FFQ

CC, Case Control; AES, Androgen Excess Society; PRL, Prolactin; TD, Thyroid Dysfunction; NCAH, Nonclassic 21-hydroxylase deficiency; CD, cushing’s disease; HTN, Hypertention; DM, Diabetes Mellitus; OCP, Oral Contraceptive Pills; CVD, Cardiovascular Disease; HA, Hyperandrogenism; CS, cushing’s syndrome; CAH, Congenital Adrenal Hyperplasia; FFQ, Food Frequency Questionnaire; USDAQ, United States Department of Agriculture (USDA) Food Security Questionnaire; 7DFQ, 7 Days Food Diary Questionnaire.

Among the included studies, two studies used the Androgen Excess Society (AES) criteria for diagnosing PCOS. One study used a clinical diagnosis, while the remaining four studies used the Rotterdam criteria. The dietary intake assessment tool varied among the studies, with four using the Food Frequency Questionnaire (FFQ), one using the United States Department of Agriculture (USDAQ), and another using the 7 Days Food Diary Questionnaire (7DFQ).

The number of participants with PCOS ranged from 99 to 347, while the number of controls ranged from 38 to 291 across the included studies. The eligibility criteria considered in each study are listed in **Table 2**. The quality assessment of the case-control studies is presented in **Table 3**. Two studies fulfilled 10 out of the 12 assessment criteria, while four studies fulfilled 9 out of the 12 criteria. All studies fulfilled at least 9 out of the 12 assessment criteria, indicating a high level of quality across the studies.

**Table 3:** Quality of the included case-control studies.

Criteria	Shishehgar et al. (22)	Hosseini et al. (19)	Badri-Fariman et al. (20)	Rajaeieh et al. (23)	Altieri et al. (24)	Z Sharkesh et al. (21)
1. Was the research question or objective in this paper clearly stated and appropriate?	Y	Y	Y	Y	Y	Y
2. Was the study population clearly specified and defined?	Y	Y	Y	Y	Y	Y
3. Did the authors include a sample size justification?	Y	Y	Y	Y	Y	Y
4. Were controls selected or recruited from the same or similar population that gave rise to the cases (including the same timeframe)?	Y	Y	Y	Y	Y	Y
5. Were the definitions, inclusion and exclusion criteria, algorithms or processes used to identify or select cases and controls valid, reliable, and implemented consistently across all study participants?	Y	Y	Y	Y	Y	Y
6. Were the cases clearly defined and differentiated from controls?	Y	Y	Y	Y	Y	Y
7. If less than 100 percent of eligible cases and/or controls were selected for the study, were the cases and/or controls randomly selected from those eligible?	N	N	N	N	N	N
8. Was there use of concurrent controls?	Y	Y	Y	Y	Y	Y
9. Were the investigators able to confirm that the exposure/risk occurred prior to the development of the condition or event that defined a participant as a case?	N	N	N	Y	Y	N
10. Were the measures of exposure/risk clearly defined, valid, reliable, and implemented consistently (including the same time period) across all study participants?	Y	Y	Y	Y	Y	Y
11. Were the assessors of exposure/risk blinded to the case or control status of participants?	N	N	N	N	N	N
12. Were key potential confounding variables measured and adjusted statistically in the analyses? If matching was used, did the investigators account for matching during study analysis?	Y	Y	Y	Y	Y	Y

#### Main findings

Out of the six case-control studies that were included in study, four of them measured overall dairy consumption. Of these, two studies reported lower dairy consumption in individuals with PCOS compared to non-infected individuals, while the other two studies reported no significant difference. One study examined the consumption of high-fat (p=0.065) and low-fat (p=0.530) dairy products separately, but there was no significant difference between the two groups. The findings from the case-control studies, including those related to dairy and cheese consumption, are presented in **Table 4**.

**Table 4:** Comparison of dairy consumption in PCOS and healthy women in case-control studies.

					Findings		
ID	Author	Dairy Type	Unit	Duration		Association direction	Description
1	Shishehgar et al. (22)	• Low fat dairy • High fat dairy	g/day	Past 12 months	NS NS	- -	-No significant difference between PCOS G. and control G. regarding consumption of Low fat dairy [Median (IQR): 206.42(54.76-351.83) Vs. 187.03(101.10-327.19) g/day, respectively; P value = 0.530] and High fat dairy(g/day) [73.06(13.77-233.41) vs. 108.23(33.11-240) g/day, respectively; P value = 0.065)]
2	Hosseini et al. (19)	Dairy products	Score: 0-10	Past 12 months	+	Inverse	-PCOS G. had a lower mean score compared to control G. (Mean (SD): (4.8 (2.6) vs. 6.0 (2.7), respectively; P value< 0.001)
3	Badri-Fariman et al. (20)	Dairy products	g/day	Past 12 months	+	Inverse	-PCOS G reported a lower dairy consumption compared to control G ((Mean (SD): 2.52 (1.58) vs. 5.69 (1.17), respectively; P value< 0.001)
4	Rajaeieh et al. (23)	-milk(overall) -Total dairy products -Skim milk -Low-fat milk -Whole milk -Cocoa milk -Other milks -Total milk -Cream -Ice cream -Low−fat yogurt -High−fat yogurt -Total yogurt -Dough -Curd -Cheese	g/day	Past 12 months	Milk(overall): + Others: NS	Direct Others: -	-No significant association between total score of dairy products and odds of PCOS G in univariate and multivariate models. -They only found a significant direct association between milk intake (g/day) and odds of PCOS after adjusting for confounding factors (OR (95% CI): …. P = 0.028).
5	Altieri et al. (24)	-Cheese -Ice cream -Low-fat milk and yogurt -Whole milk and yogurt	g/day	Past 7 days	Cheese: + Others: NS	Direct Others: -	PCOS G. compare to control G. had a higher mean of cheese consumption (Mean (SD): 59·2(37·1) Vs. 49·6(28·8); P value= 0·049), respectively; but no significant differences was observed between two groups regarding other assessed items.
6	Zirak Sharkesh et al. (21)	Dairy products	g/day	Past 12 months	NS	–	No significant difference between PCOS G and control G groups regarding mean dairy products consumption [Mean (SD): 122.23 (97.45) Vs. 139.29 (83.55) g/day, respectively; P value = 0.038]

NS, Not Significant; IQR, Interquartile Range; SD, Standard Deviation.

In one study, milk consumption was found to be significantly higher in individuals with PCOS after adjusting for confounding factors (P=0.028), but there was no significant difference between the two groups in terms of milk consumption by milk type. In five of the studies reviewed, dairy consumption in the last 12 months was measured using a scale of g/day, while in one study, it was measured over the last 7 days using the same scale. Additionally, the amount of dairy consumption was measured using a scale of g/day in all studies reviewed, except for one study in which it was measured by scoring on a scale of 0-10.

Two studies examined cheese consumption separately. In one study, there was a significant difference in cheese consumption between individuals with PCOS and controls. In the other study, there was no significant difference observed.

### Interventional studies

#### Description

No studies were identified that specifically examined the effectiveness of reducing or increasing the amount or type of dairy consumption separately. However, four interventional studies were found that evaluated the effect of simultaneously reducing dairy and starch consumption (12, 15–17). The eligible women in these studies were between 18 and 45 years of age. In these studies, participants consumed a high-fat, low-carbohydrate liquid meal (HSFLM) consisting of 8 floz (237 ml) of chocolate Ensure (Abbott Laboratories, Chicago, Illinois), which contained a total of 6 g of fat (1 g of saturated fat (SFA), 3 g of polyunsaturated fat, and 2 g of monounsaturated fat), 40 g of carbohydrate, and 9 g of protein. To increase the proportion of SFA and make the liquid meal an SFA-rich high-fat meal, 32 g of butter, 5 g of coconut oil, and 19 g of palm oil were added to the chocolate Ensure. After the addition of dietary fat and lecithin, the total grams of fat in the high-fat meal equaled 56 g, which constituted 68% of total calories.

All four studies were conducted in the United States and included a total of 51 participants, with a range of 10 to 24 participants per study. In all four studies, PCOS was diagnosed using the Rotterdam criteria. The eligibility criteria for each study are summarized in **Table 5**. The quality assessment of the before-and-after intervention studies with no control group is outlined in **Table 6**. All studies fulfilled 10 out of the 12 assessment criteria, indicating a high level of quality across the studies.

**Table 5:** Characteristics of the Included Interventional Studies. (Randomization: no).

ID	Author	Country	Sample size (Loss to FU)	Final sample size	Age(year) Mean ± SD/Range	PCOS diagnosis	Eligibility criteria	Intervention	Duration (week)
1	Pohlmeier et al. (2014) (12)	USA	13 (3)	10	29.6 ± 4.6	Rotterdam criteria	Overweight and obese women with at least one polycystic ovary by ultrasound, without adrenal enzyme defects, tumors, DM2, late onset 21-hydroxylase deficiency, medical condition requiring supervision, and oligomenorrhea, and not using insulin sensitizers, OCP, cyclic progesterone for one month prior to the study.	A low starch/low dairy diet	8
2	Phy et al. (2015) (15)	USA	28 (4)	24	29.8 ± 4.0	Rotterdam criteria	Overweight and obese women with at least one polycystic ovary by ultrasound, without adrenal enzyme defects, tumors, DM2, late onset 21-hydroxylase deficiency, medical condition requiring supervision, and oligomenorrhea, and not using insulin sensitizers, OCP, cyclic progesterone for one month prior to the study.	A low starch/low dairy diet	8
3	Phy et al. (2013 a) (16)	USA	21 (3)	18	18 to 45	Rotterdam criteria	Women with PCOS aged 18 to 45 years	A low starch/low dairy diet	8
4	Phy et al. (2013 b) (17)	USA	21 (3)	18	18 to 45	Rotterdam criteria	Women with PCOS aged 18 to 45 years	A low starch/low dairy diet	8

FU, Follow Upp; DM, Diabetes Mellitus.

**Table 6:** Quality of the Included Interventional Studies (14).

Criteria	Pohlmeier et al. (12)	Phy et al. (2015) (15)	Phy et al. (2013 a) (16)	Phy et al. (2013 b) (17)
1. Was the study question or objective clearly stated?	Y	Y	Y	Y
2. Were eligibility/selection criteria for the study population prespecified and clearly described?	Y	Y	Y	Y
3. Were the participants in the study representative of those who would be eligible for the test/service/intervention in the general or clinical population of interest?	Y	Y	Y	Y
4. Were all eligible participants that met the prespecified entry criteria enrolled?	Y	Y	Y	Y
5. Was the sample size sufficiently large to provide confidence in the findings?	Y	Y	Y	Y
6. Was the test/service/intervention clearly described and delivered consistently across the study population?	Y	Y	Y	Y
7. Were the outcome measures prespecified, clearly defined, valid, reliable, and assessed consistently across all study participants?	Y	Y	Y	Y
8. Were the people assessing the outcomes blinded to the participants’ exposures/interventions?	N	N	N	N
9. Was the loss to follow-up after baseline 20% or less? Were those lost to follow-up accounted for in the analysis?	Y	Y	Y	Y
10. Did the statistical methods examine changes in outcome measures from before to after the intervention? Were statistical tests done that provided p values for the pre-to-post changes?	Y	Y	Y	Y
11. Were outcome measures of interest taken multiple times before the intervention and multiple times after the intervention (i.e., did they use an interrupted time-series design)?	N	N	N	N
12. If the intervention was conducted at a group level (e.g., a whole hospital, a community, etc.) did the statistical analysis take into account the use of individual-level data to determine effects at the group level?	Y	Y	Y	Y

#### Main findings

**Figure 2:**
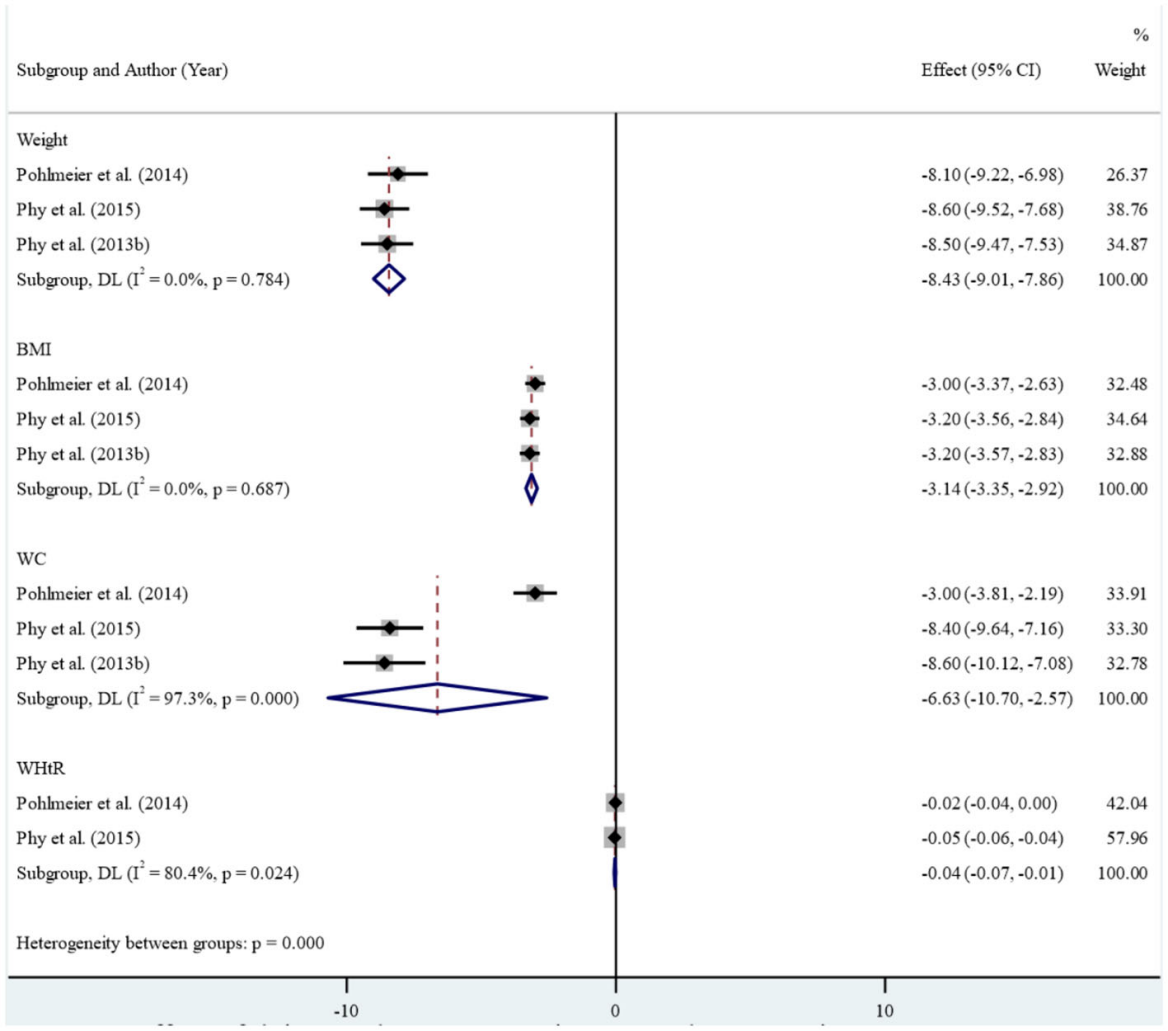
Effect of low dairy-low starch diet on anthropometric measurements.

**Figure 3:**
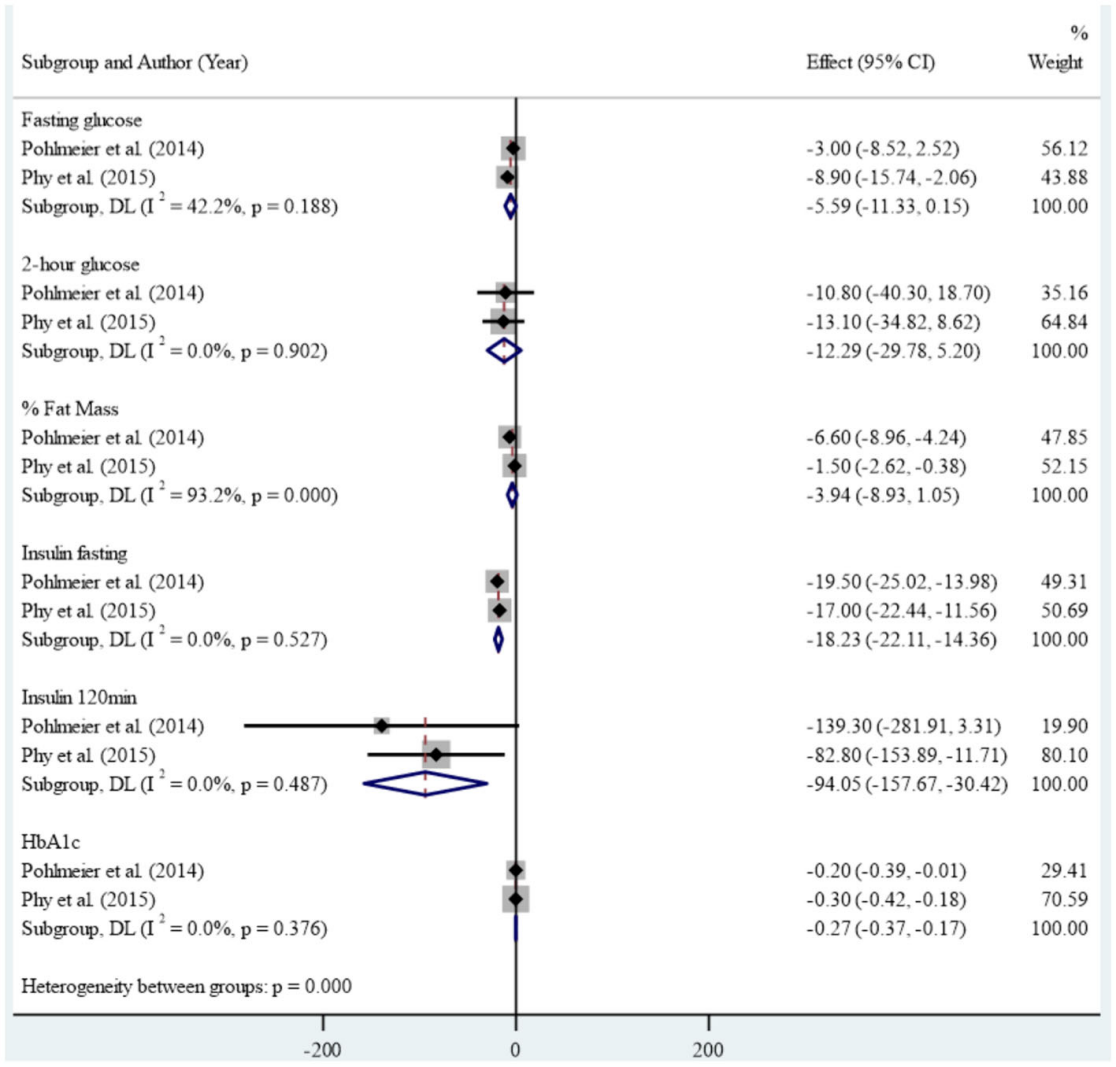
Effect of low-diary-low starch diet on blood glucose tests.

**Figure 4:**
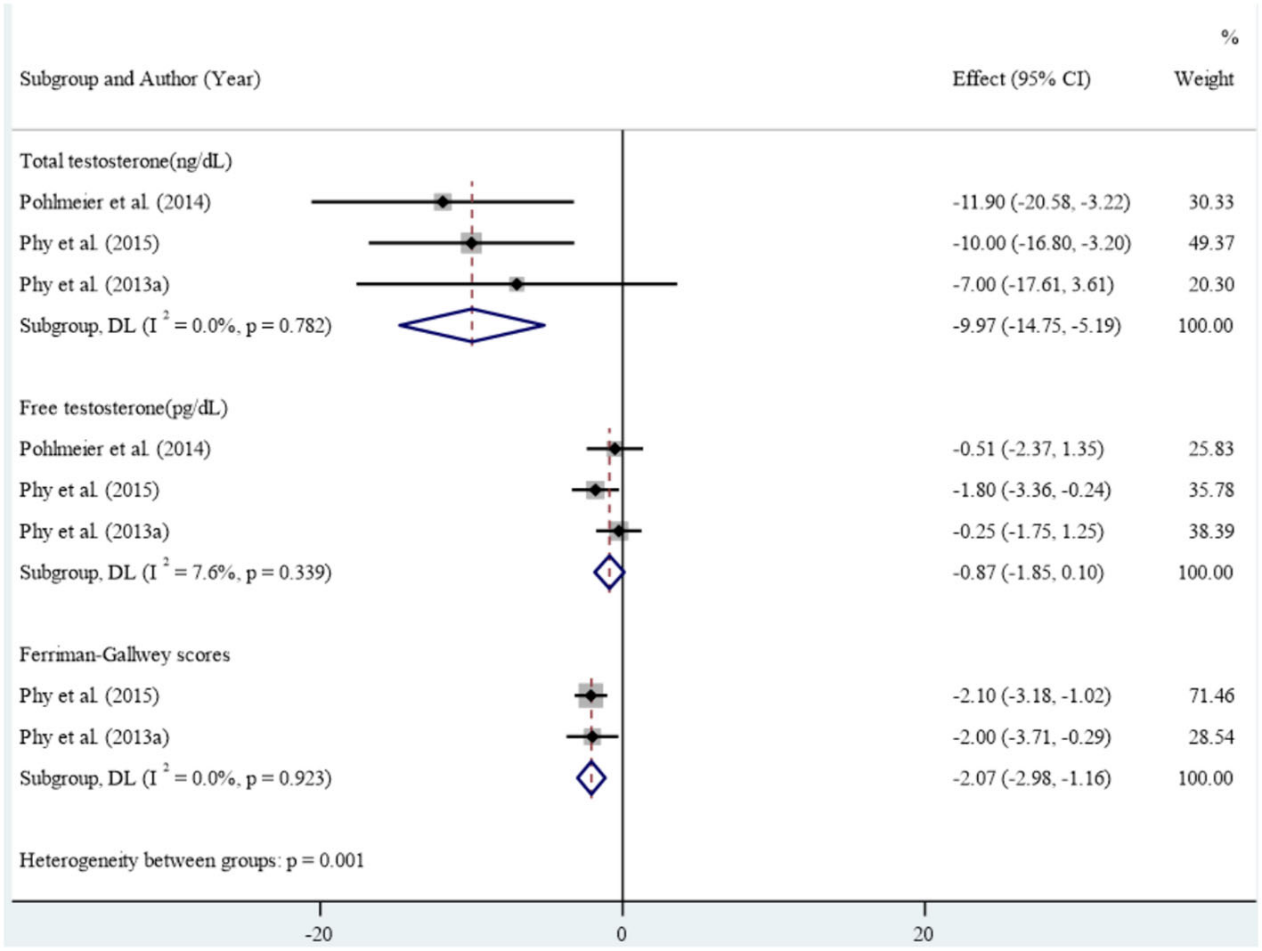
Effect of low dairy-low starch diet on Ferrhman-Gallwey score and testosterone level.

In the clinical studies included in the meta-analysis, high levels of heterogeneity were observed in the association between reducing dairy product consumption and the mean of anthropometric, blood glucose, Ferryman-Gallwey score, and testosterone level components (p-value<0.001). Therefore, a random-effect model was used to analyze the data. After reducing dairy product consumption, the results demonstrated statistically significant reductions in mean weight (SMD: -8.43 (95% CI: -9.01, -7.86)), BMI (-3.14 (95% CI: -3.35, -2.92)), WC (-6.63 (95% CI: -10.70, -2.57)), and WHtR (-0.04 (95% CI: -0.07, -0.01)) compared to pre-intervention levels. (**Figure 2**). Furthermore, a notable association was found between reducing dairy product consumption and the mean levels of fasting insulin (-18.23 (95% CI: -22.11, -14.36)), insulin at 120 minutes (-94.05 (95% CI: -157.67, -30.42)), and HbA1c (-0.27 (-0.37, -0.17)). However, no significant reduction was observed in the mean levels of fasting glucose, 2-hour glucose, and percentage of fat mass before and after the intervention (**Figure 3**). Regarding the Ferryman-Gallwey score and testosterone level components, there was a statistically significant decrease in the mean of total testosterone (-9.97 (95% CI: -14.75, -5.19)) and Ferryman-Gallwey score (-2.07 (95% CI: -2.98, -1.16)) after the intervention compared to before. However, there was no significant association observed between the two groups in terms of the mean of free testosterone after the intervention (**Figure 4**).

### Cross-sectional studies

#### Description

This cross-sectional study assessed the dietary quality of 100 women diagnosed with PCOS. All participants met the Rotterdam criteria for PCOS diagnosis and were within the reproductive age range of 18–45 years. Anthropometric measurements, including weight, height, WC, BMI, and hip circumference (HC) were obtained. Dietary intake was evaluated using the Brazilian Healthy Eating Index – Revised (BHEI-R), where higher scores reflect better dietary quality (18). The quality of this study was assessed and outlined in **Table 7**. It fulfilled 10 out of the 14 assessment criteria, indicating a high level of quality.

**Table 7:** Quality of the Included Cross-Sectional Studies (14).

Criteria	Rodrigues AM
1. Was the research question or objective in this paper clearly stated?	Y
2. Was the study population clearly specified and defined?	Y
3. Was the participation rate of eligible persons at least 50%?	Y
4. Were all the subjects selected or recruited from the same or similar populations (including the same time period)? Were inclusion and exclusion criteria for being in the study prespecified and applied uniformly to all participants?	Y
5. Was a sample size justification, power description, or variance and effect estimates provided?	Y
6. For the analyses in this paper, were the exposure(s) of interest measured prior to the outcome(s) being measured?	Y
7. Was the timeframe sufficient so that one could reasonably expect to see an association between exposure and outcome if it existed?	N
8. For exposures that can vary in amount or level, did the study examine different levels of the exposure as related to the outcome (e.g., categories of exposure, or exposure measured as continuous variable)?	Y
9. Were the exposure measures (independent variables) clearly defined, valid, reliable, and implemented consistently across all study participants?	Y
10. Was the exposure(s) assessed more than once over time?	N
11. Were the outcome measures (dependent variables) clearly defined, valid, reliable, and implemented consistently across all study participants?	Y
12. Were the outcome assessors blinded to the exposure status of participants?	N
13. Was loss to follow-up after baseline 20% or less?	Y
14. Were key potential confounding variables measured and adjusted statistically for their impact on the relationship between exposure(s) and outcome(s)?	Y

The prevalence of overweight and obesity was 30.0% and 66.0%, respectively, with 90.0% exhibiting increased visceral fat mass, indicating a heightened risk of metabolic complications. Additionally, 64.0% were categorized as sedentary, while 36.0% were classified as less active (18).

#### Main finding

The mean BHEI-R score was 56.1 ± 12.0 points, with 56.0% of participants reporting an inadequate diet and 44.0% reporting a diet that needs improvement’. The BHEI-R score showed negative correlations with measures of obesity, including BMI (r = 0.248; P = 0.013), body weight (r = 0.220; P = 0.028), and WC (r = 0.278; P = 0.005).

## Discussion

In this study, we reviewed 11 studies that met our inclusion criteria out of the 1,313 citations reviewed. These studies consisted of six case-control studies, four clinical trials, and one cross-sectional study. The meta-analysis of the clinical trials studies revealed statistically significant improvements in various clinically relevant measures when compared to the pre-intervention period. These included reductions in weight, BMI, waist circumference, waist-to-height ratio, fasting insulin, insulin at 120 minutes, HbA1c, and Ferryman-Gallwey score. However, there were no significant changes observed in fasting glucose, 2-hour glucose, percent of fat mass, and mean free testosterone levels between the two groups following the intervention.

### Main findings

In general, our research suggests that there is controversy regarding association between dairy consumption and PCOS occurrence. Some studies, such as Hosseini et al. and Badri-Fariman et al., detected an inverse correlation between dairy consumption and PCOS (19, 20), while others, such as Zirak Sharkesh et al. and Shishehgar et al., found no significant relationship (21, 22). Rajaeieh et al.’s study showed a direct relationship between milk consumption and PCOS, while Altieri et al. found a direct relationship with cheese consumption (23, 24).

Overall, it is impossible to conclude that dairy consumption is a risk factor for PCOS, and further research is needed to determine whether reducing dairy consumption can improve PCOS symptoms.

All four interventional studies reviewed found that a low-dairy/low-starch diet had a positive impact on anthropometric factors and glycemic control, as demonstrated in the studies conducted by Pohlmeier et al., Phy et al., and Phy et al. b (1, 16, 17). Additionally, the diet was shown to have positive effects on testosterone levels in the studies conducted by Phy et al., Phy et al. a, and Pohlmeier et al. (1, 15, 17) Overall, these studies suggest that a low-dairy/low-starch diet may be effective in improving laboratory and anthropometric factors in patients with PCOS. The results suggest that reducing the intake of dairy and starch in the diet may lead to beneficial impacts on laboratory measures in people with polycystic ovary syndrome.

Janiszewska et al.’s study showed that including whole milk and dairy products in the diet of women with polycystic ovary syndrome could be beneficial due to their positive effect on the risk of developing type 2 diabetes mellitus in women (25). However, this finding was not supported by experimental and clinical studies, which instead showed that reducing dairy and starch consumption had a positive effect on glycemic control, as demonstrated in our study.

Temperament as the final homogeneous quality results from the interactions of opposite qualities- heat, coldness, moisture, and dryness - the four philosophical elements when they combine and form the composite materials of the world. Based on this, everything, including food and medicine and even conditions such as weather and climate, has its temperament, which is determined by the change it imposes on human temperament. Due to this effect, their temperament can cause human disease or be used to maintain health or treat diseases in different people. Large groups of diseases are caused by bad temperaments provoked in a part or the whole human body. Therefore, disease prevention or treatment may be facilitated by recognizing and treating the underlying temperament disorder. The temperament of the whole body or specific organs has been observed by sages with scenarios of signs and symptoms accumulated over the centuries (26, 27).

Iranian medical texts describe milk as consisting of three parts: fat, water, and cheese (carbohydrates and protein). The fat part of milk has a hot and dry temperament, while the cheese part has a cold and dry temperament, and the water has a cold and wet temperament. This means that as milk fat content increases, its temperament becomes warmer, and if the fat content is low, its coldness increases. For example, high-fat milk and dairy products are warmer than low-fat options, and buttermilk and yogurt are colder than milk (28, 29).

In traditional Iranian medicine, PCOS is considered a disease with a cold and wet temperament (30). Therefore, it is important to carefully observe the avoidance of dairy products with a cold and wet temperament in people with PCOS (31).

Chavarro’s study suggested that high intake of low-fat dairy foods may increase the risk of anovulatory infertility, while intake of high-fat dairy foods may decrease this risk. Lactose, the main carbohydrate in milk and dairy products is not believed to affect fertility within the usual range of intake levels in humans (32, 33).

Shishehgar’s study found that PCOS cases consumed less high-fat dairy than controls, but the difference was not statistically significant (22). However, in other studies, the temperament of the consumed dairy product has not been considered, which may be a reason for the different and contradictory results.

In observational studies, in most cases, the consumption of dairy products has been lower in the polycystic ovary syndrome group, but in interventional studies, reducing dairy consumption has had a positive effect on improving anthropometric and laboratory factors.

To make more accurate and informed decisions regarding the consumption of dairy products in PCOS patients, it is important to conduct both interventional and descriptive studies that take into account the temperament of dairy products. This may help to clarify the relationship between dairy consumption and PCOS symptoms and provide more specific recommendations regarding the types and amounts of dairy products that are safe and beneficial for PCOS patients.

### Limitations

One limitation of our study was the absence of intervention studies that solely investigated the impact of reducing or increasing dairy consumption. All four interventional studies that we analyzed focused on the effect of simultaneously reducing dairy and starch consumption. Another limitation was the lack of studies that considered the type of dairy products consumed and the temperament of patients with PCOS. These factors are important in assessing the impact of dairy products on the disease and could influence the results of the studies.

## Conclusion

In summary, the findings of this systematic review show limited evidence about the association between dairy consumption and PCOS. The interventional studies suggest that reducing dairy consumption in combination with reducing starch intake may improve some anthropometric and metabolic measures in PCOS women, including weight, BMI, waist circumference, Waist-to-Height Ratio, insulin fasting, insulin 120 minutes, HbA1c. However, more research with larger sample sizes and more diverse populations is needed to fully understand the effects of dairy consumption on PCOS.

## Data Availability

The original contributions presented in the study are included in the article/supplementary material. Further inquiries can be directed to the corresponding author.
